# Mechanical Properties and Fabrication of Bioinspired Cactus Spine Microneedles

**DOI:** 10.3390/bios16090488

**Published:** 2026-09-03

**Authors:** Hongru Liu, Xiang Long, Qiumeng Sun, Shixiong Wu, Zhishan Yuan

**Affiliations:** 1 School of Electro-Mechanical Engineering, Guangdong University of Technology, Guangzhou 510006, China; liuhongru@mails.gdut.edu.cn (H.L.); 2112401481@mail2.gdut.edu.cn (X.L.); qiumeng.sun12@gmail.com (Q.S.); wushixionggdut@163.com (S.W.); 2 Guangdong Provincial Key Laboratory of Minimally Invasive Surgical Instruments and Manufacturing Technology, Guangdong University of Technology, Guangzhou 510006, China; 3 State Key Laboratory for High Performance Tools, Guangdong University of Technology, Guangzhou 510006, China

**Keywords:** cactus spines, bioinspired microneedles, microstructure, metal micromolds, mechanical testing

## Abstract

Microneedle-based transdermal drug delivery enables painless and efficient drug administration but is limited by insufficient mechanical strength and high insertion forces. Inspired by the efficient penetration capability of cactus spines, this study investigated the microstructure and biomechanics of natural cactus spines and bioinspired microneedles. Finite element analysis showed that a groove width of 50 μm produced the highest stress and strain. Solid bioinspired microneedles were fabricated by 3D printing, while dissolvable hyaluronic acid, chitosan, and gelatin microneedles were prepared using femtosecond laser-fabricated titanium molds and replica molding. Optimized laser parameters generated micropores approximately 500 μm deep. Mechanical tests showed insertion forces of 60–100 mN for solid microneedles, with the 50 μm groove design exhibiting the highest value. Among dissolvable microneedles, gelatin displayed the greatest mechanical strength, whereas hyaluronic acid demonstrated the best overall potential for transdermal drug delivery.

## 1. Introduction

Microneedle-based transdermal drug delivery technology enables direct transport of therapeutic agents to the epidermal or dermal layers by penetrating the stratum corneum barrier. This approach offers advantages including high delivery efficiency, operational convenience, painlessness, and reduced risk of infection [1,2,3], effectively overcoming the limitations of conventional administration routes such as low oral bioavailability [4] and the severe pain associated with subcutaneous injections [5]. Nevertheless, conventional microneedles commonly face a trade-off between mechanical strength and insertion force. Silicon microneedles exhibit high strength but are prone to brittle fracture [6], metal microneedles are difficult to fabricate [7], dissolvable microneedles demonstrate good biocompatibility yet suffer from insufficient hardness and susceptibility to bending [6], and hydrogel microneedles similarly encounter challenges related to poor mechanical performance [8]. Hollow microneedles represent another important class, offering distinct advantages for active drug delivery and interstitial fluid extraction via an internal lumen [7,9]; however, the introduction of a central bore substantially reduces the effective cross-sectional area and creates stress concentrations at the inner wall, rendering hollow designs more susceptible to buckling, torsion, and brittle fracture during skin insertion compared with their solid counterparts [9]. Consequently, the development of novel microneedle structures that combine high strength, low insertion force, and excellent biocompatibility has emerged as a critical challenge in the field of transdermal drug delivery.

A comparative analysis of the literature further reveals that the mechanical performance of microneedles varies considerably across different studies, depending on geometric and material parameters. For example, Römgens et al. [10] reported that the insertion force of single solid microneedles increased linearly from approximately 20 to 167 mN as the tip diameter increased from 5 to 37 μm, emphasizing the critical role of tip sharpness in reducing penetration resistance. In a broader geometric comparison, Davis et al. [11] demonstrated that insertion forces ranged from 0.1 to 3 N for various microneedle designs, scaling proportionally with the interfacial area of the needle tip. Additionally, systematic evaluations of needle geometry have indicated that conical microneedles typically exhibit lower insertion forces compared to pyramidal and flat-tipped configurations [12]. These discrepancies among reported results highlight that the balance between mechanical strength and insertion force is highly sensitive to structural design, necessitating a biomimetic optimization strategy to unify these contrasting outcomes.

As naturally occurring, highly efficient, low-damage penetration structures, cactus spines have been widely adopted as biomimetic prototypes for microneedle design in recent years. Cactus spines possess a distinctive tapered asymmetric geometry, featuring a sharp tip that gradually widens toward the base. This morphological configuration effectively exploits the Laplace pressure difference to achieve directional, self-propelled droplet transport [13,14,15,16,17]. Inspired by this mechanism, researchers have developed various cactus spine-inspired microneedle arrays. For instance, Jiang’s team at Sun Yat-sen University [18,19] utilized laser direct writing technology to fabricate bioinspired spines with backward micro-barbs and hierarchical microchannels for ultrafast water transport and efficient fog harvesting. Other studies [20] employed a magnetic particle-assisted molding (MPAM) method to prepare PDMS-based conical microneedle arrays, demonstrating that the tip morphology can be precisely controlled by adjusting the PDMS-to-magnetic-particle ratio. Kharbikar et al. [9] drew inspiration from natural micron-scale structures such as cactus spines and combined multiphysics simulation with the Bosch process to fabricate high-aspect ratio hollow silicon microneedle arrays for transdermal delivery of antiemetic drugs to manage chemotherapy-induced nausea and vomiting. In 2021, Son et al. [21] developed a cactus spine-inspired sweat-collection patch that utilizes wedge-shaped wettability-patterned channels and hierarchical micro/nanostructured surfaces to induce unidirectional Laplace pressure, enabling efficient sweat transport and continuous biochemical monitoring even in vertically oriented wear. Furthermore, Liu’s team at Jilin University [22] integrated the energy gradient mechanism of cactus spines with the microgroove structure of rice leaves to develop a bioinspired micro-spine array (BMSA) that achieves directional water droplet transport at speeds as high as 237.42 mm/s, opening new avenues for multifunctional applications including oil–water separation and fog collection. These biomimetic structures not only preserve the mechanical penetration advantages of cactus spines, but also incorporate hydrophilic/hydrophobic surface gradients, thereby enabling dual functionality of penetration and substance transport.

**Figure 1:**
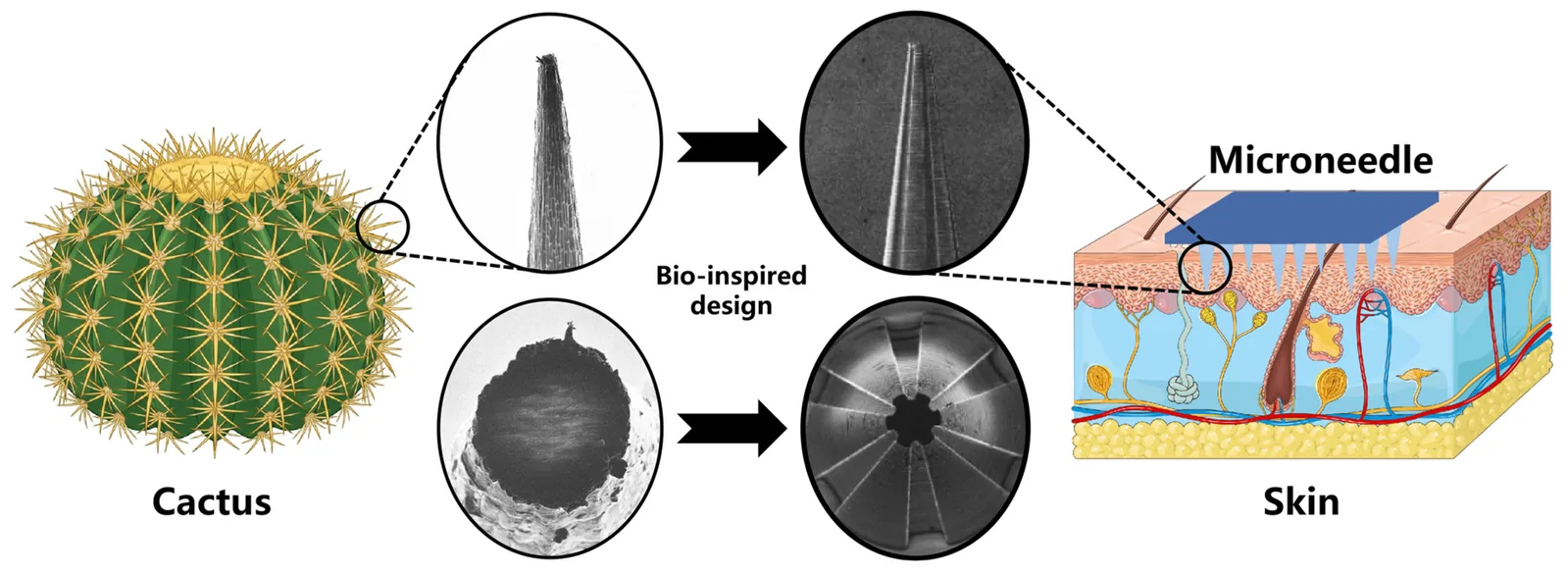
Schematic diagram of bioinspired cactus spine microneedle penetration into skin tissue.

Although researchers have successfully utilized cactus spine microstructures to achieve functions such as directional droplet transport and fog harvesting, investigations into the biomechanical performance of these structures during skin penetration remain relatively scarce. In light of this, the present study adopts the cactus spine as a biomimetic prototype (Figure 1). CT scanning and helium ion scanning electron microscopy (He-SEM) were employed to observe the surface microstructure of natural cactus spines, with in-depth investigation of surface groove morphology and optimization of critical structural parameters such as groove width. Bioinspired cactus spine solid microneedles with varying dimensions were fabricated via 3D printing technology. Femtosecond laser processing was utilized to prepare metal molds, followed by replica molding to produce bioinspired cactus spine dissolvable microneedles. A mechanical analysis model for the bioinspired microneedles was established, and a custom-designed multifunctional mechanical testing system was constructed to evaluate the mechanical properties of both natural spines and microneedles during penetration into PDMS skin simulants. This work aims to establish a theoretical and technological foundation for the practical application of microneedles in transdermal drug delivery, interstitial fluid extraction, and microsensing.

## 2. Experimental Section

### 2.1. Theoretical Analysis of Microneedle Forces

During skin penetration, microneedles primarily experience bending and compressive forces. To simplify the analysis, the microneedle is modeled as a cantilever beam fixed at one end, and linear mathematical equations are established:

(1) $$y=(\frac{L}{D_{1}-D_{2}})(-2x+D_{1})$$

where y∈[0,L], $x\in [\frac{D_{2}}{2},\frac{D_{1}}{2}]$, and $D_{1}>2D_{2}$.

The bending force induces lateral deflection of the microneedle. The maximum bending force is related to the bending section modulus of the circular cross-section:

(2) $$F_{b}=\frac{\sigma_{1}W}{l}$$

where $\sigma_{1}$ is the bending strength of the microneedle, F is the bending force the microneedle can withstand, l is the length of the microneedle, and W is the bending section modulus of the circular cross-section (frustum of a cone). For a conical microneedle, $W=\frac{\pi d^{3}}{32}$, l=L−y, d=2x.

(3) $$F_{b}=\frac{\sigma_{1}\times \frac{\pi }{32}\times {\left(2x\right)}^{3}}{L-(\frac{L}{D_{1}-D_{2}})(-2x+D_{1})}$$

The critical cross-section is located at $x=\frac{3}{4}D_{2}$, where the maximum bending force is $F_{b}=\frac{27\pi \sigma_{1}D_{2}2(D_{1}-D_{2})}{128L}$.

Axial compression is the primary force responsible for stratum corneum penetration. According to column buckling theory, the critical compressive force is related to the material’s elastic modulus, needle length, and tip diameter:

(4) $$F_{a}=\frac{\pi^{3}E{\left(D_{1}-D_{2}\right)}^{2}x^{4}}{16L^{2}{\left(2x-D_{2}\right)}^{2}}$$

when $x\in [\frac{D_{2}}{2},\frac{D_{1}}{2}]$, and E is the elastic modulus of the material. The maximum is obtained at $x=D_{2}$:$F_{a}=\frac{\pi^{3}ED_{2}2{\left(D_{1}-D_{2}\right)}^{2}}{16L^{2}}$. The bending stress that the microneedle can withstand is:

(5) $$\sigma_{2}=\frac{F_{a}}{A}$$

where A is the tip cross-sectional area.

The microneedle strength design must simultaneously satisfy both bending and compressive strength requirements. Theoretically, the pressure required to penetrate human skin is 3.183 MPa. When the pressure sustained by the microneedle exceeds this value, the design criterion is satisfied. Substituting σ_2_ = 3.183 MPa into the above equation yields the compressive force required for skin penetration:

(6) $$F_{a}=3.183+\frac{\pi D_{2}2}{4}$$

When penetration occurs at an angular deviation, the additional bending force must also be considered:

(7) $$F_{b}=\frac{1}{2}\sin\alpha (3.183+\frac{\pi D_{2}2}{4})$$

The maximum stress the microneedle can sustain is:

(8) $$\sigma_{max}=\sigma_{1}+\sigma_{2}\leq \left[\sigma \right]$$

where σ is the allowable stress of the material. Combining (1) and (7):

(9) $$\sigma_{max}=3.183[\frac{16L}{27(D_{1}-D_{2})}\sin\alpha +1]\leq \left[\sigma \right]$$

In summary, the transverse force on the microneedle is closely related to the needle length, base diameter, tip diameter, and material properties.

### 2.2. Mechanical Simulation Analysis

The explicit dynamics module of Workbench 19.0 software was employed for simulation analysis.

Skin exhibits anisotropic, nonlinear, viscoelastic, and diverse characteristics. In the present simulation, it was simplified as an isotropic, incompressible elastic material [23,24], modeled using the Neo-Hookean and Mooney–Rivlin models:

(10) $$W=\sum_{i,j=0}^{\infty }C_{ij}{\left(I_{1}-3\right)}^{i}{\left(I_{2}-3\right)}^{j}$$

where $C_{ij}$ are material constants, and $I_{1}$ and $I_{2}$ are the first and second invariants of the strain tensor.

(11) $$W=C_{10}(I_{1}-3)$$

(12) $$W=C_{10}+C_{01}(I_{2}-3)$$

where $C_{10}$ and $C_{01}$ are constants, with $C_{10}\approx 7.6∼26$ MPa [25]. In evaluating skin strength parameters (tensile, compressive, and shear strength), reasonable assumptions were made based on actual conditions, and appropriate numerical values were selected [26,27,28], treating the skin as an anisotropic elastic body (Table 1).

The simulation procedure included model establishment, model import, material parameter assignment, mesh generation, fixed constraint application, force application, and analytical solution.

### 2.3. Microneedle Fabrication

Microneedles were fabricated using 3D printing technology. First, a three-dimensional model of the microneedle array was established using SolidWorks 2023 software and exported as an STL file. The model was then imported into a nanoArch S140 (BMF Precision Tech Inc., Chongqing, China) micro-stereolithography (DLP) printer with a layer thickness setting of 10 μm. Photosensitive resin was added to the resin tank, and layer-by-layer exposure and curing were performed according to preset parameters to complete the printing of the microneedle array. After printing, the samples were removed from the build platform, cleaned with anhydrous ethanol to remove uncured resin, and subjected to secondary UV curing to enhance structural stability. Finally, the microneedles were characterized for dimensions and morphology, yielding the 3D-printed microneedle samples.

A 5-Axis Laser Processing Machine (Xi’an Micromach Photon Manufacture Technology Co., Ltd., Xi’an, China) was used to machine micropore molds on medical-grade titanium alloy surfaces. Characterization was performed using an automatic grinding machine (Xi’an Nagashima Seiko Machinery Co., Ltd., Xi’an, China), a dual-lens measuring instrument (Marcel Aubert SA, Ipsach, Switzerland), and a high-definition CCD measuring microscope (Kunshan Gaopin Precision Instrument Co., Ltd., Suzhou, China). Preliminary experiments were conducted with a processing power of 6 W, frequency of 20 kHz, 50 scanning cycles, scanning speed of 600 mm/s, and spacing of 20 μm.

Dissolvable microneedles were prepared using a replica molding technique. The selection of hyaluronic acid (HA), chitosan, and gelatin as the matrix materials was based on their distinct and complementary physicochemical and biological properties, which are widely recognized as representative natural polymers for transdermal drug delivery applications [1,8,29]. Hyaluronic acid, a glycosaminoglycan naturally present in human connective tissue, offers excellent biocompatibility, biodegradability, and non-immunogenicity, making it an ideal candidate for minimally invasive skin penetration without eliciting adverse immune responses. Chitosan, a polycationic polysaccharide derived from chitin, is characterized by its rapid degradation, inherent antimicrobial activity, and immunomodulatory properties, which can facilitate wound healing and enhance drug permeation through the skin [30]. Gelatin, a denatured derivative of collagen, exhibits relatively high mechanical strength and good film-forming ability, which contributes to the structural integrity of microneedles during insertion [31]. By maintaining all other processing parameters constant, this study aimed to systematically compare the effects of these three distinct polymer compositions on the mechanical performance and penetration capability of the bioinspired cactus spine microneedles. The titanium alloy molds were ultrasonically cleaned for 1 h and soaked in ethanol for 30 min. Subsequently, pre-degassed molding solutions were added, and the molds were subjected to ultrasonic oscillation for 30 min to promote complete filling of the micropores. Vacuum drying and curing were then performed, followed by demolding to obtain the microneedle patches. Hyaluronic acid, chitosan, and gelatin were used as the three materials for dissolvable microneedle preparation, with all other processing parameters maintained constant to compare the effects of different materials on microneedle performance.

### 2.4. Mechanical Performance Testing

Silicone rubber base (Dow Corning SYLGARD 184, Midland, MI, USA) and curing agent were mixed at a weight ratio of 50:1, degassed, cast, and cured to prepare PDMS skin simulant. PDMS was selected as the skin simulant for several reasons. First, it offers excellent batch-to-batch reproducibility and standardized mechanical properties, thereby avoiding the ethical constraints and inherent biological variability associated with ex vivo human or animal skin samples [32,33]. Second, PDMS is optically transparent, which facilitates real-time visual observation of the microneedle penetration process and post-penetration morphological analysis [32]. Third, the mechanical properties of PDMS—particularly its Young’s modulus—can be readily tuned by adjusting the base-to-curing-agent ratio and curing conditions, allowing its stiffness to be tailored to approximate the mechanical response of human dermis [34,35].

Prior to mechanical testing, the homogeneity of the PDMS skin simulant was verified through a two-step quality-control protocol. (i) Visual and microscopic inspection: cross-sectional slices were cut from multiple locations of the cured PDMS block and examined under an optical microscope to confirm uniform thickness and the absence of air bubbles, cracks, or filler segregation. (ii) Mechanical consistency assessment: indentation tests were performed at five randomly selected positions on the sample surface using the same universal testing machine. The resulting force–displacement curves exhibited a relative standard deviation of less than 5% in elastic modulus across all measured points, confirming uniform mechanical properties throughout the simulant [36].

Nevertheless, the inherent limitations of PDMS as a skin surrogate must be explicitly acknowledged. PDMS is a homogeneous, isotropic elastomer; although its Young’s modulus can be tuned to approximate the stiffness of human dermis, it cannot replicate the anisotropic, nonlinear, viscoelastic, and stratified architecture of natural skin [37]. Consequently, within the scope of this work, the PDMS skin simulant is employed strictly as a preliminary engineering screening tool with the following intended purposes: (1) to compare the relative insertion forces of bioinspired microneedles with varying groove widths and of dissolvable microneedles composed of different materials under standardized conditions; (2) to evaluate whether the microneedles undergo structural failure modes such as fracture, buckling, or excessive bending during penetration; and (3) to establish reproducible baseline mechanical data free from biological variability. It is emphasized that the penetration depths and force–displacement profiles obtained in PDMS are not interpreted as being directly representative of in vivo human skin penetration [34,37]. Future studies will validate the mechanical trends observed herein using ex vivo porcine skin, which is established as an excellent model for human skin [33].

A custom-designed multifunctional mechanical testing system was employed for microneedle penetration testing. During the test, microneedles were vertically inserted into the PDMS skin simulant at a speed of 1.0 mm/s, and both insertion and extraction forces were recorded. Testing was performed on natural cactus spines, four groove-width variants (30, 40, 50, and 60 μm) of bioinspired grooved solid microneedles, and dissolvable microneedles made of hyaluronic acid (Freda, Jinang, China), chitosan (Aladdin, Shanghai, China), and gelatin (Aladdin, Shanghai, China). For mechanical characterization, individual microneedles were tested to isolate the intrinsic effect of groove geometry; array samples were employed for morphological evaluation. After testing, optical microscopy and scanning electron microscopy (SEM) were used to observe morphological changes before and after penetration, and deformation conditions were documented.

## 3. Results and Discussion

### 3.1. Microstructural Characterization of Cactus Spines and Structural Design of Bioinspired Microneedles

**Figure 2:**
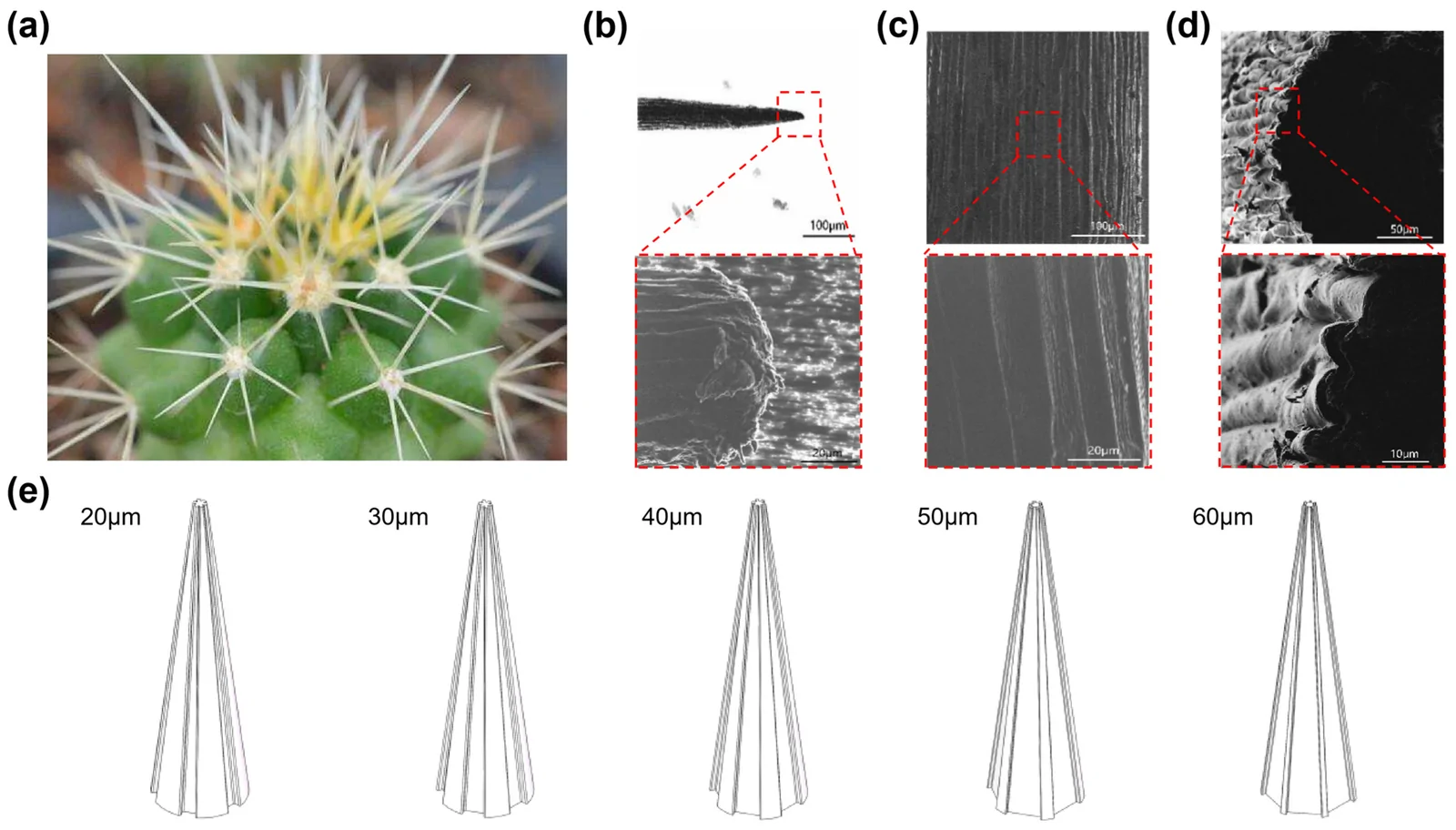
(**a**) Optical photograph of golden barrel cactus; (**b**) SEM image of cactus spine tip; (**c**) SEM image of cactus spine surface; (**d**) SEM image of cactus spine side view; (**e**) Bioinspired cactus spine microneedles with varying groove widths.

Echinocactus grusonii was selected as the biomimetic object, and its surface microstructure was characterized using helium ion scanning electron microscopy (He-SEM) (Figure 2a). The characterization revealed that the cactus spine surface exhibits orderly, smooth, strip-like groove structures. The spine tip is not an ideal conical shape, but rather possesses a certain curvature. Groove widths range from approximately 1–5 μm near the tip to 5–20 μm away from the tip, with tip diameters of approximately 2–10 μm (Figure 2b–d). Groove width and depth measurements were obtained using Image-Pro Plus software. By varying the groove depth and width parameters, bioinspired microneedle structures with different configurations were designed, and three-dimensional models were established using SolidWorks. Considering the measurement data, factors influencing mechanical performance, and subsequent fabrication feasibility, the final structural parameters for the bioinspired microneedles were determined as follows: tip groove width of 2–6 μm, base groove width of 20–60 μm, groove depth of 10 μm, and ridge arc radius of 2 μm (Figure 2e).

### 3.2. Mechanical Simulation of Bioinspired Cactus Spine Microneedles

**Figure 3:**
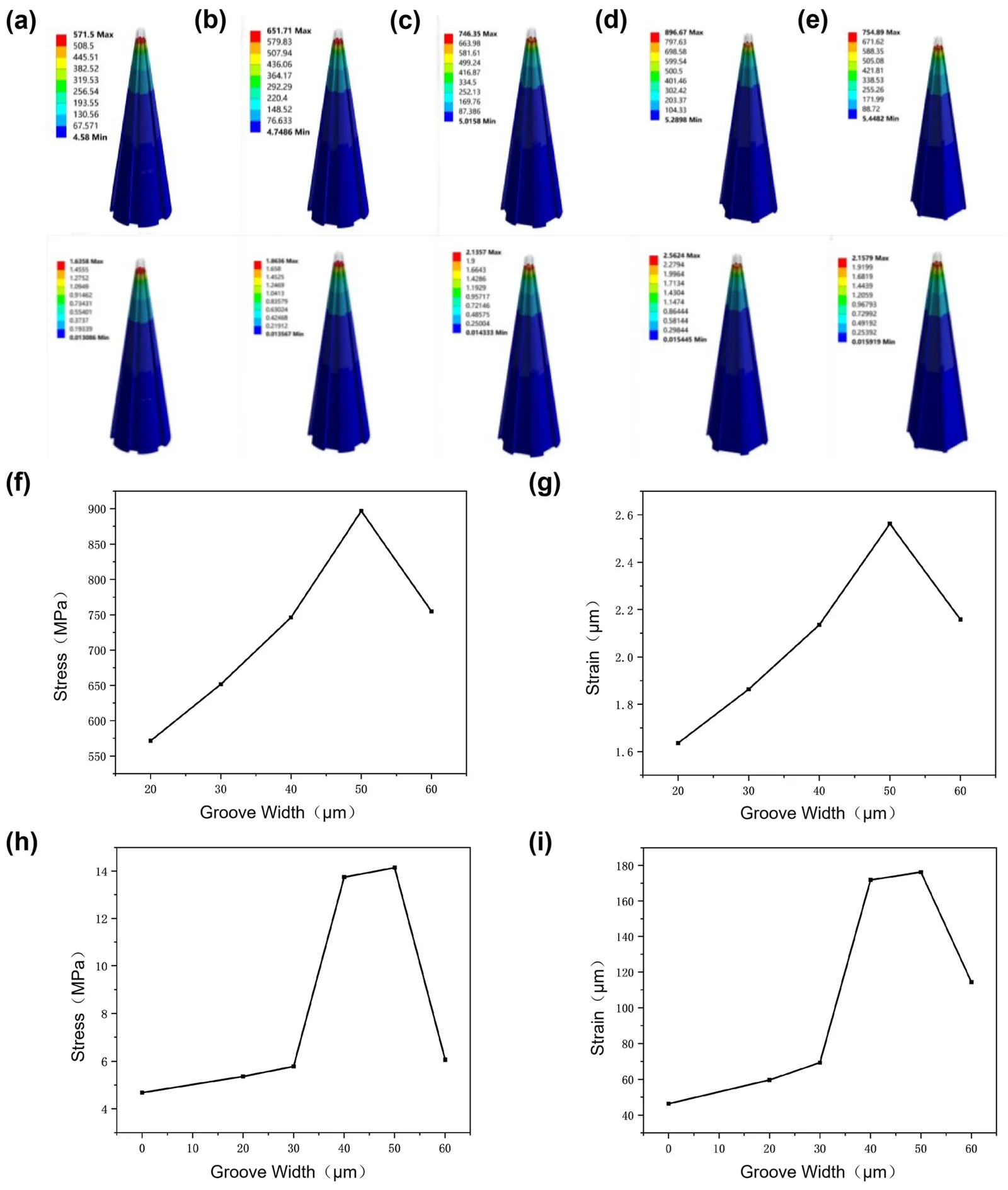
(**a**–**e**) Equivalent stress (**top**) and equivalent strain (**bottom**) diagrams for bioinspired cactus spine microneedles with varying groove widths: (**a**) 20 μm; (**b**) 30 μm; (**c**) 40 μm; (**d**) 50 μm; (**e**) 60 μm; (**f**) Equivalent stress variation curve for bioinspired microneedles with different groove widths; (**g**) Equivalent strain variation curve for bioinspired microneedles with different groove widths; (**h**) Equivalent stress variation curve for skin under different groove widths; (**i**) Equivalent strain variation curve for skin under different groove widths.

The explicit dynamics module of Workbench 19.0 software was employed to simulate the penetration process of bioinspired cactus spine microneedles into skin. A conventional conical microneedle was used as a control, which exhibited a stress of 4.68 MPa and a strain of 46.38 μm upon skin insertion. Subsequently, the mechanical performance of five bioinspired microneedle variants with different groove widths (20, 30, 40, 50, and 60 μm) was simulated. By comparing the maximum equivalent stress and equivalent strain that both the microneedle and the skin could withstand, the design criterion—that the microneedle must penetrate the stratum corneum without fracturing—was evaluated. In the simulation, the large end of the microneedle was fixed, and a force of 3.813 MPa was applied in the vertical direction at the small end (Figure 3a–e).

To further elucidate the localized mechanical behavior, the von Mises stress distribution at the tip region and groove features was calculated with enhanced mesh refinement. The results revealed significant stress concentration at the tip apex, where the maximum von Mises stress reached 754.69 MPa for the 50 μm groove width variant (Figure 3f). Notably, secondary stress concentration was observed at the groove edges and valley intersections. The 50 μm design exhibited the most pronounced localized stress at both the tip and groove root, correlating with its highest insertion force and optimal penetration depth.

The simulation results indicated that as groove width increased, both the stress and strain experienced by the microneedle and the skin initially increased and then decreased (Figure 3f,i). At a groove width of 20 μm, the maximum skin stress was 5.36 MPa with a strain of 59.68 μm, and the stress required to penetrate the stratum corneum was 1.10 MPa. At 30 μm, the skin stress reached 5.78 MPa with a strain of 69.44 μm, enabling penetration into the epidermal layer. At 40 μm, the stress increased markedly to 13.74 MPa with a strain of 171.79 μm, achieving a penetration depth of 140 μm, sufficient to traverse the stratum corneum and reach the dermis. At 50 μm, both stress and strain peaked at 14.14 MPa and 176.24 μm, respectively, with a penetration depth of 180 μm and a stratum corneum penetration stress of 4.83 MPa. At 60 μm, the stress dropped sharply to 6.06 MPa with a strain of 114.31 μm, yielding a penetration depth of 110 μm. The stresses sustained by the microneedle itself were substantially greater than those experienced by the skin in all cases, satisfying the strength requirements.

**Figure 4:**
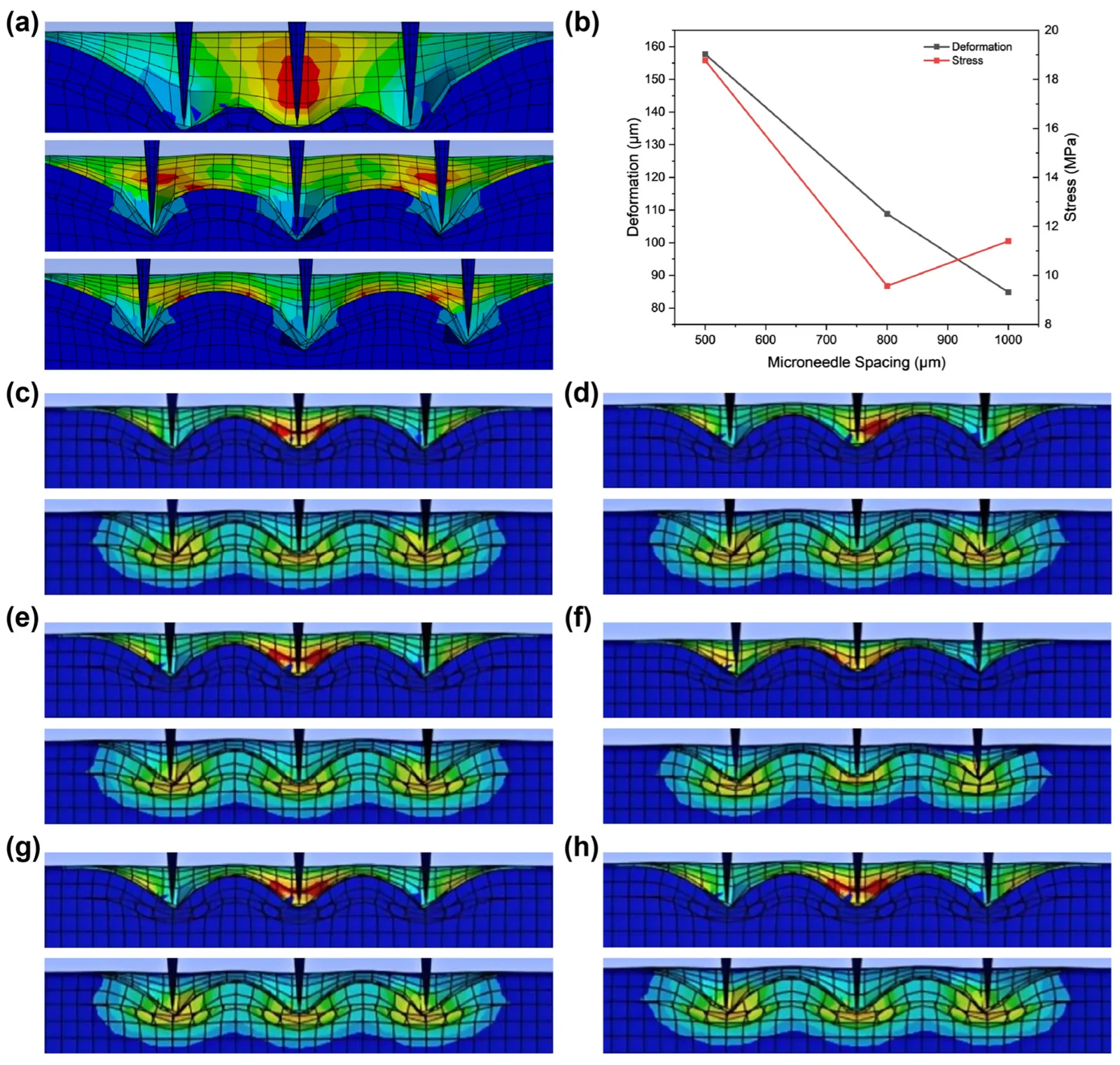
(**a**) Schematic diagram of microneedle arrays with base spacings of 200 μm (**top**), 500 μm (**middle**), and 800 μm (**bottom**) penetrating the skin simulation; (**b**) Deformation and stress of the skin caused by the bed of nails effect when microneedle arrays with base spacings of 200 μm, 500 μm, and 800 μm penetrate the skin; (**c**–**h**) Equivalent stress (**top**) and equivalent strain (**bottom**) results for skin: (**c**) Conventional microneedle; (**d**) 20 μm bioinspired microneedle; (**c**) 30 μm bioinspired microneedle; (**e**) 40 μm bioinspired microneedle; (**f**) 50 μm bioinspired microneedle; (**g**) 60 μm bioinspired microneedle.

Subsequently, the penetration process of conventional microneedle arrays and bioinspired cactus spine microneedle arrays into skin was simulated. To investigate the effect of microneedle spacing, array configurations comprising 3 × 3 microneedles with center-to-center distances of 500 μm, 800 μm, and 1000 μm were modeled. At 500 μm spacing, overlapping stress fields from adjacent microneedles amplified the bed-of-nails effect. At 800 μm spacing, optimal penetration efficiency was achieved with minimal inter-needle mechanical interference. At 1000 μm spacing, the bed-of-nails effect diminished, requiring higher overall applied force for complete array penetration. The results indicated that 800 μm spacing provided an optimal balance between array density and mechanical performance (Figure 4a,b).

The simulation results demonstrated that although the bed-of-nails effect produced by the bioinspired microneedle arrays differed slightly from that of the conventional arrays, the overall trends were similar, and all groups of microneedles effectively penetrated the stratum corneum and reached the target dermal depth. Both the conventional and bioinspired microneedle arrays formed distinct stress concentration zones at the tip–contact regions during penetration, and the deformation zones of the skin tissue were predominantly localized around the needle tips (Figure 4c,h).

### 3.3. Fabrication of Bioinspired Cactus Spine Microneedles

**Figure 5:**
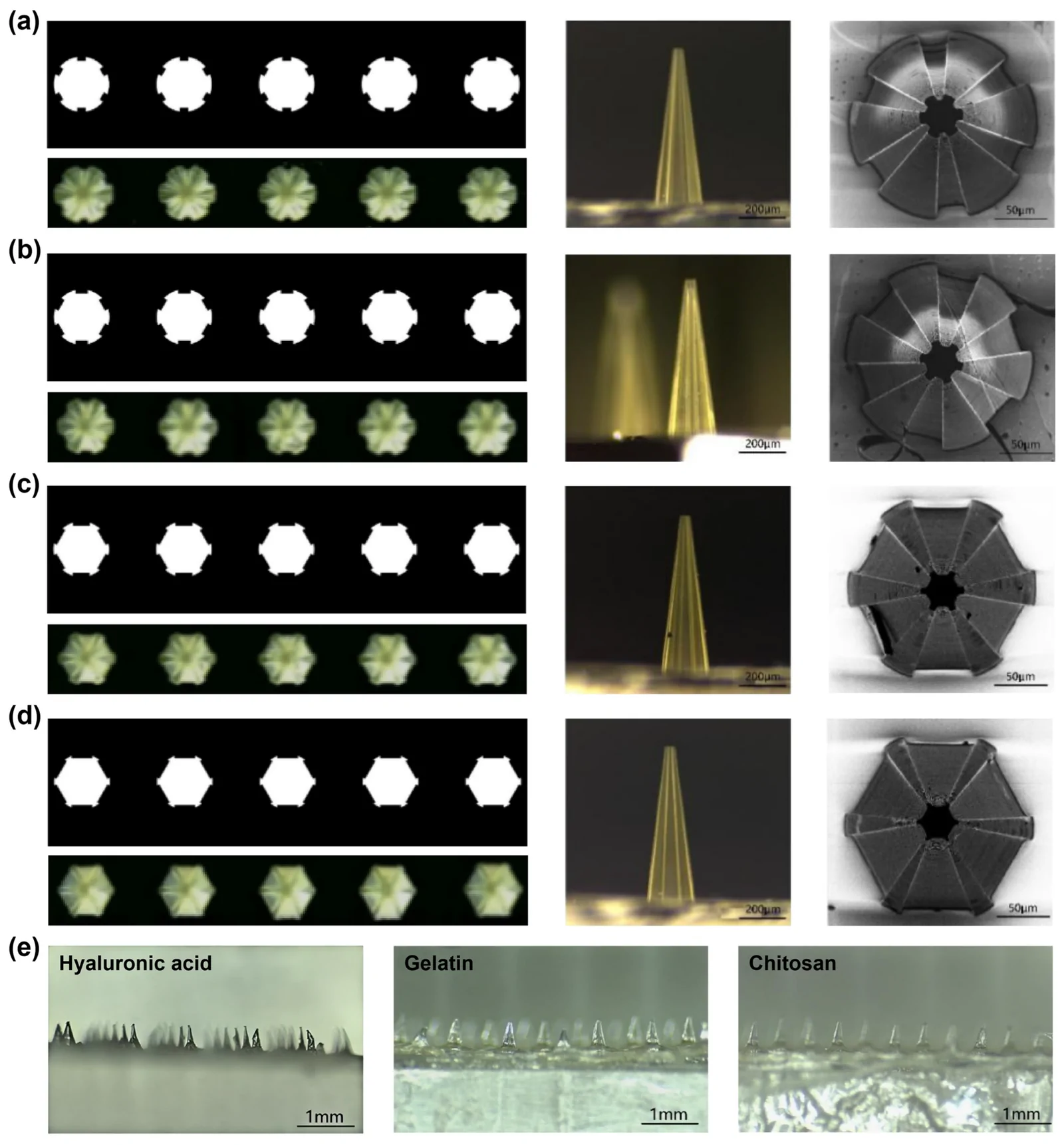
(**a**) Optical image, side view, and top view of 3D-printed bioinspired microneedle with 30 μm groove width; (**b**) 40 μm groove width; (**c**) 50 μm groove width; (**d**) 60 μm groove width. (**e**) Optical images of bioinspired cactus spine dissolvable microneedle arrays.

Bioinspired resin microneedles with groove widths of 30, 40, 50, and 60 μm were successfully fabricated via 3D printing technology. The printed microneedles exhibited smooth, orderly groove structures with dimensions that closely matched the CAD models (Figure 5a–d). Concurrently, titanium alloy molds were processed using laser ablation, and dissolvable microneedles composed of three different materials—hyaluronic acid, chitosan, and gelatin—were prepared via replica molding (Figure 5e). Hyaluronic acid, which naturally occurs in the human body, offers excellent biocompatibility, biodegradability, and non-immunogenicity. Chitosan is a natural polymer characterized by rapid degradation and immunomodulatory properties. Gelatin exhibits relatively high mechanical strength. Optical microscopy observations indicated that chitosan microneedles most effectively replicated the mold geometry.

**Figure 6:**
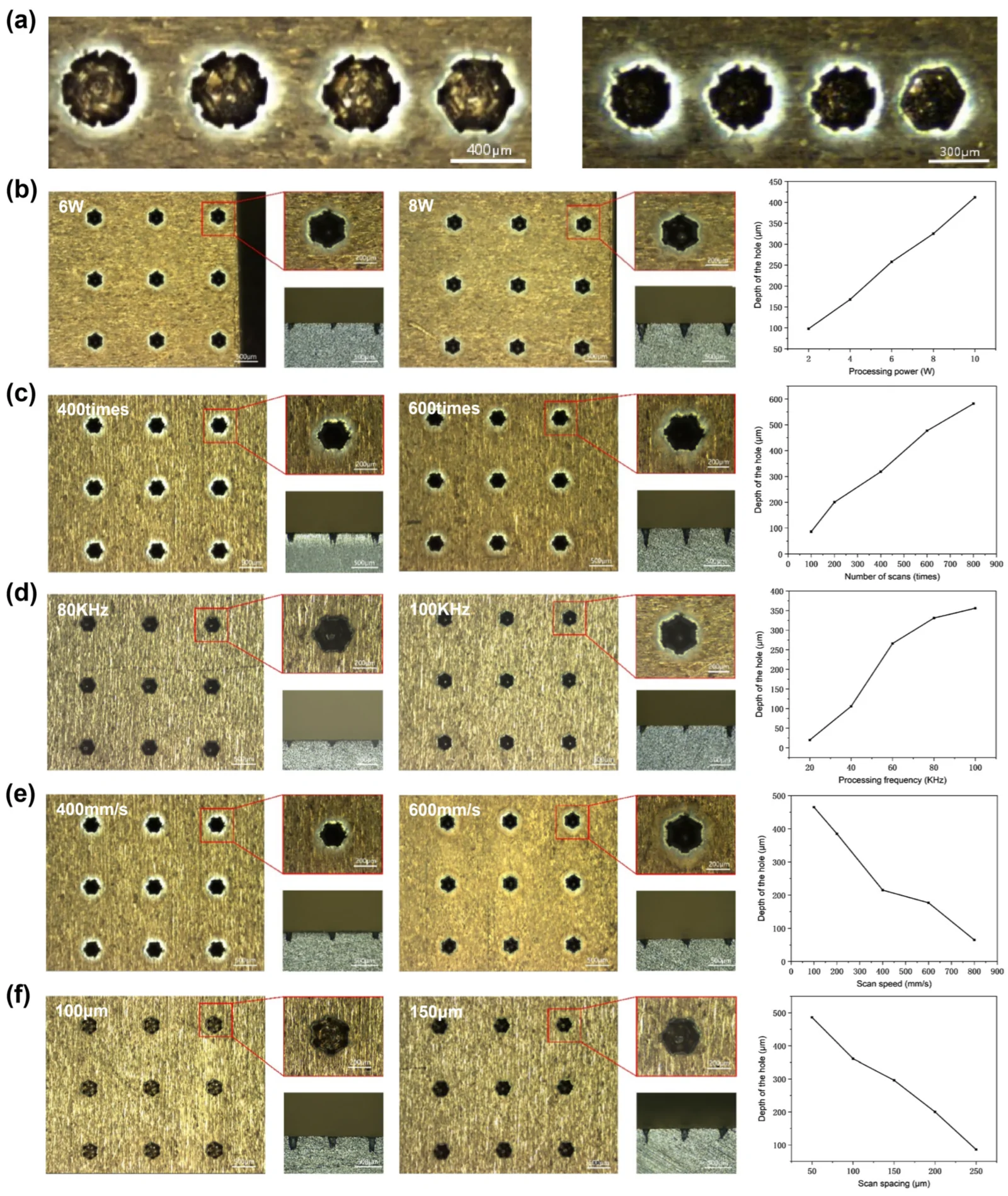
(**a**) Processing outcomes for pore diameters of 400 μm (left) and 300 μm (right); (**b**) Effect of processing power on surface quality and depth of micropores with 50 μm groove width; (**c**) Effect of scanning cycles on surface quality and depth of micropores with 50 μm groove width; (**d**) Effect of processing frequency on surface quality and depth of micropores with 50 μm groove width; (**e**) Effect of scanning speed on surface quality and depth of micropores with 50 μm groove width; (**f**) Effect of scanning spacing on surface quality and depth of micropores with 50 μm groove width. All array images in (**b**–**f**) display 3 × 3 micropore arrangements. Scale bars: 500 μm for the main array images, 200 μm for the magnified top-view insets, and 500 μm for the cross-sectional views.

In this study, femtosecond laser processing was employed to fabricate micropore molds on medical-grade titanium alloy surfaces. It was observed that larger pore diameters yielded better molding quality (Figure 6a). Using pore surface quality and pore depth as evaluation criteria, the effects of five processing parameters—laser power, number of scanning cycles, frequency, scanning speed, and scanning spacing—on mold quality were systematically investigated.

Single-variable experimental results revealed the following: With regard to laser power, under conditions of 400 mm/s scanning speed, 200 cycles, 20 kHz frequency, and 20 μm spacing, the optimal surface morphology was achieved at 8 W. Pore depth increased from 98 μm to 412 μm as power increased from 2 W to 10 W; however, at 10 W, the surface contour became indistinct (Figure 6b). Regarding scanning cycles, under conditions of 8 W power, 400 mm/s speed, 20 kHz frequency, and 20 μm spacing, optimal surface quality and ideal depth were obtained at 400 or 600 cycles (Figure 6c). For processing frequency, 100 kHz yielded a pore depth of 356 μm with the most favorable surface contour and no residual material within the pores (Figure 6d). Concerning scanning speed, under conditions of 8 W power, 200 cycles, 20 kHz frequency, and 20 μm spacing, pore depth decreased from 465 μm to 65 μm as speed increased from 100 mm/s to 800 mm/s (Figure 6e). For scanning spacing, the optimal surface morphology was achieved at 100–150 μm; larger spacing resulted in reduced depth due to insufficient overlap of the spiral scanning path and variations in Gaussian energy distribution (Figure 6f). Based on the comprehensive evaluation of these factors, the optimal processing parameters were determined as follows: pulse energy of 1 mJ, power of 6 W, scanning speed of 400 mm/s, spacing of 150 μm, frequency of 100 kHz, and 400 scanning cycles, yielding micropores with depths of approximately 400–500 μm and excellent surface quality.

### 3.4. Mechanical Properties of Bioinspired Cactus Spine Microneedles

**Figure 7:**
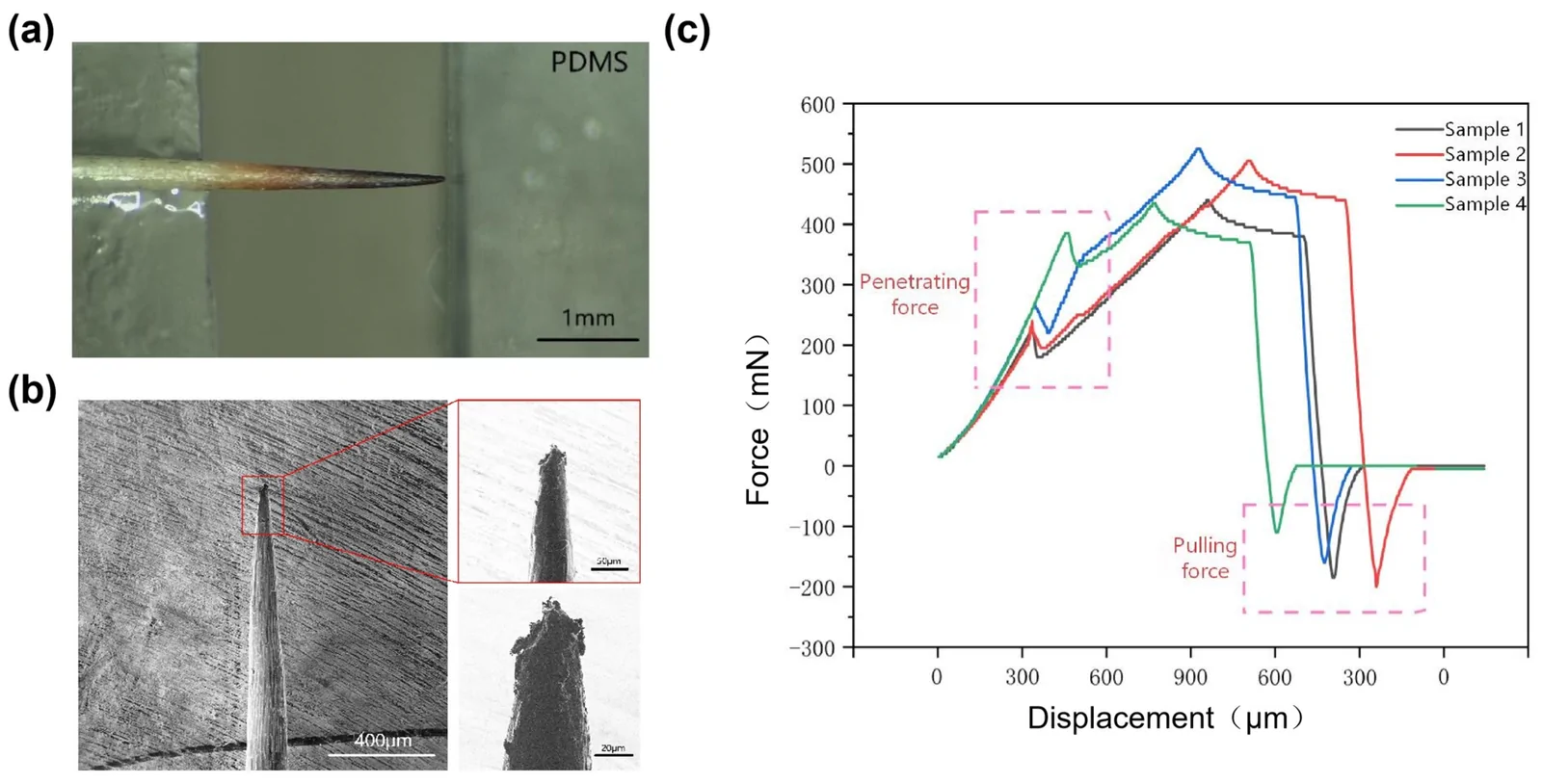
(**a**) Optical image of cactus spine penetrating PDMS; (**b**) SEM images of cactus spine tip and surface after withdrawal from PDMS; (**c**) Mechanical testing curves for four natural cactus spines.

A custom-designed multifunctional mechanical testing system was utilized to evaluate the biomechanical properties of natural cactus spines. Four spines with similar dimensions and morphology and smooth surfaces were selected and sequentially mounted on the testing platform for penetration into PDMS skin simulant (Figure 7a). Repeated testing of the four natural spines (Figure 7c) revealed insertion forces ranging from approximately 200 to 370 mN and extraction forces ranging from approximately 110 to 230 mN. All spines successfully penetrated the skin simulant without fracturing. Post-testing SEM micrographs (Figure 7b) showed that the natural spines exhibited only PDMS residue at the tips, with smooth surface microstructures and no evidence of structural damage, indicating suitability for drug delivery applications.

**Figure 8:**
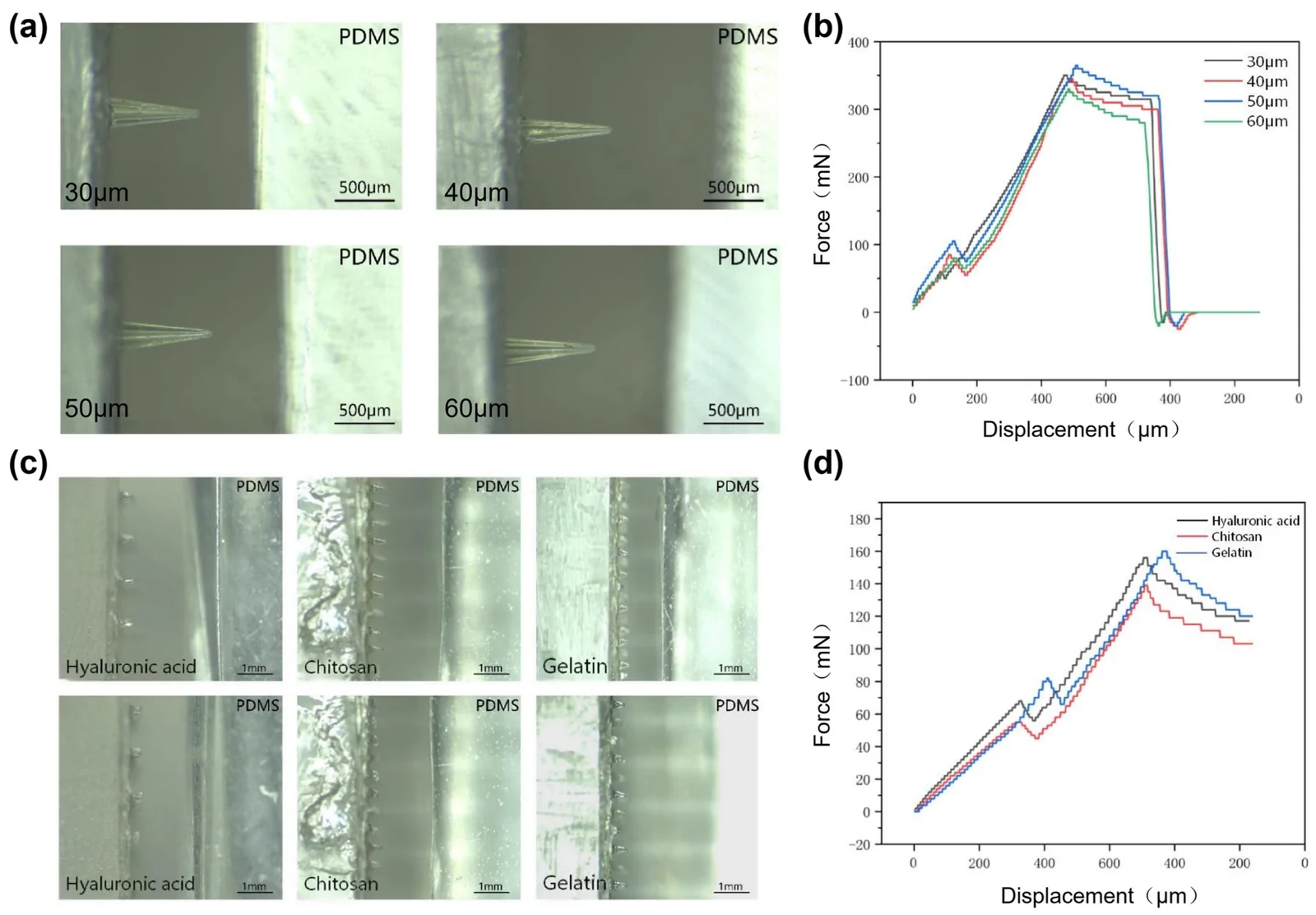
(**a**) Optical images of solid bioinspired cactus spine microneedles with different groove widths penetrating PDMS; (**b**) Mechanical curves of four solid bioinspired cactus−spine microneedles penetrating PDMS; (**c**) Optical images of bioinspired cactus−spine dissolvable microneedles before (top) and after (bottom) PDMS penetration; (**d**) Mechanical testing curves of three bioinspired cactus spine dissolvable microneedles.

The same testing protocol was applied to four bioinspired microneedle variants with different groove widths. The results demonstrated that all four solid bioinspired microneedles possessed sufficient strength to penetrate the skin simulant without fracturing, with insertion forces ranging from 60 to 100 mN. Among these, the microneedle with a groove width of 50 μm exhibited the largest insertion force, which was 10–15 mN higher than the other three variants and 40–100 mN lower than that of natural spines (Figure 8a,b).

Dissolvable microneedles with a groove width of 50 μm were prepared from three biocompatible materials—hyaluronic acid, chitosan, and gelatin—using the replica molding technique, and their mechanical properties were investigated. The observed differences in mechanical performance among the three dissolvable microneedles can be attributed to the intrinsic material properties of each polymer. Gelatin microneedles exhibited the largest insertion force with minimal bending deformation, which is consistent with the higher mechanical rigidity of gelatin derived from its collagen-based triple-helix structure. In contrast, chitosan microneedles displayed the smallest insertion force with the greatest bending deformation, likely due to the relatively brittle nature of pure chitosan and its limited solubility under physiological conditions, which has been reported to restrict its mechanical robustness in microneedle applications [30]. Hyaluronic acid microneedles demonstrated an intermediate insertion force and moderate deformation, reflecting a balance between the viscoelastic nature of HA and its lower mechanical strength compared to gelatin [38]. Notably, all three types of dissolvable microneedles remained intact without fracturing during penetration, and their insertion forces were significantly lower than those of the solid 3D-printed microneedles (Figure 8c,d). Among the three polymers, hyaluronic acid microneedles exhibited the most balanced overall performance, combining adequate mechanical strength for successful skin penetration with favorable biocompatibility and degradation characteristics, aligning with its established role as a versatile platform for transdermal drug delivery [1,8].

## 4. Conclusions

This study presents a comprehensive investigation of the structural design, mechanical simulation, fabrication processes, and performance characterization of cactus spine-inspired microneedles. Based on the characterization of the surface groove microstructure of natural cactus spines, design parameters were established with tip groove widths of 2–6 μm, base groove widths of 20–60 μm, and a groove depth of 10 μm. Finite element simulations revealed that, as groove width increased, both the stress and strain experienced by the microneedle and the skin initially increased and then decreased, peaking at a groove width of 50 μm. The solid microneedles fabricated via 3D printing closely matched the CAD models, although the needle tips exhibited a plateau-like morphology. For the dissolvable microneedles prepared by replica molding, the optimal femtosecond laser processing parameters for mold fabrication were determined as follows: pulse energy of 1 mJ, power of 6 W, scanning speed of 400 mm/s, spacing of 150 μm, frequency of 100 kHz, and 400 scanning cycles, yielding high-quality micropores with depths of approximately 400–500 μm. All four solid bioinspired microneedle variants successfully penetrated the PDMS skin simulant with insertion forces ranging from 60 to 100 mN without fracturing, with the 50 μm groove width variant exhibiting the highest insertion force. Among the three dissolvable microneedle materials, gelatin showed the highest insertion force, chitosan the lowest, and hyaluronic acid exhibited intermediate performance with the most balanced overall properties. Notably, all bioinspired microneedles demonstrated significantly lower insertion forces than natural cactus spines (200–370 mN).

Although the present study focused on solid and dissolvable bioinspired microneedles, the grooved cactus spine design offers a natural pathway for extension to hollow configurations. The longitudinal surface grooves could be engineered to serve as external microchannels or reinforcing ribs, potentially mitigating the mechanical penalties associated with hollowing while preserving the directional fluid transport capability inspired by natural cactus spines.

## Figures and Tables

**Table 1:** Parameters of microneedles and skin.

	Thickness/mm	Density/kg·m^−3^	Failure Strength/MPa	Elastic Modulus/MPa	Poisson’s Ration
Microneedle	—	1251.5	—	3500	0.36
Stratum corneum	0.02	1300	28.5	120	0.45
Epidermis	0.08	1230	8.4	120	0.39
Dermis	1.5	1200	7.3	5	0.38
Subcutaneous tissue layer	1.0	971	—	0.5	0.38

## Data Availability

The original contributions presented in this study are included in the article. Further inquiries can be directed to the corresponding author.
